# Metabolic dysfunction and the use of adjunct medications in type 1 diabetes

**DOI:** 10.1097/MED.0000000000000956

**Published:** 2026-03-26

**Authors:** Andrew Luk, Yee Seun Cheah, Shivani Misra, Victoria Salem

**Affiliations:** a School of Medicine, Imperial College London; b Department of Diabetes, King's College Hospital NHS Foundation Trust; c Metabolism, Digestion & Reproduction; d Department of Bioengineering, Faculty of Engineering, Imperial College London, London, UK

**Keywords:** adjunctive therapy, glucagon-like peptide-1 receptor agonists, obesity, sodium-glucose cotransporter-2 inhibitors, type 1 diabetes mellitus

## Abstract

**Purpose of review:**

Type 1 diabetes is increasingly complicated by obesity and broader metabolic dysfunction, yet there are barriers to the use of adjunctive pharmacotherapies in this population. This review evaluates the evidence for metformin, glucagon-like peptide-1 (GLP-1) receptor agonists (GLP-1RAs), and sodium-glucose cotransporter-2 (SGLT2) inhibitors (SGLT2i) in type 1 diabetes, with a focus on weight loss, glycaemic control, insulin dose requirements and safety.

**Recent findings:**

Despite advances, metformin remains the only adjunct widely endorsed in national guidelines for adults with type 1 diabetes. In clinical trials, GLP-1RAs used alongside automated insulin delivery systems demonstrate significant improvements in weight, glucose sensor time-in-range, and total daily insulin dose, without increased risk of diabetic ketoacidosis (DKA). SGLT2i produce more modest weight and HbA1c improvements, and may be associated with an increased risk of DKA, although they have a clear evidence base for independent cardiovascular benefits.

**Summary:**

There is an increasing demand by patients and desire by physicians to utilize adjunctive medications in type 1 diabetes. Many patients with type 1 diabetes who are highly likely to benefit from the weight loss and cardiorenal risk reduction effects of these drugs are denied access to them because of putative safety concerns and a dearth of clinical trial evidence in type 1 diabetes. Identifying patients with type 1 diabetes most likely to tolerate and benefit from these agents is a research priority. Real world datasets accounting for the increased off license use of these drugs offers an opportunity to rapidly develop evidence-based guidance.

## INTRODUCTION

Treatment pathways for type 1 diabetes and type 2 diabetes have historically been distinct, reflecting fundamental differences in their aetiopathogenesis. This dichotomy acknowledges that the β-cell dysfunction of type 2 diabetes sits within a broader spectrum of obesity-driven phenotypes including insulin resistance, dyslipidaemia, hypertension and generalized inflammation (henceforth referred to as “metabolic dysfunction”). In contrast, type 1 diabetes is seen as a condition of absolute insulin deficiency as the result of autoimmune β-cell destruction. Therefore, treatment for type 1 diabetes has remained almost exclusively glucocentric, with the only licenced drug treatment being insulin, and the cardinal features of metabolic dysfunction, such as insulin resistance, often overlooked. In contrast, there is increasing recognition of clinical heterogeneity in type 2 diabetes, coupled with the rapid expansion of drug classes available for its treatment, which has driven interest in precision medicine pathways for metabolic health [1,2]. The latest treatment guidelines for type 2 diabetes recommend the use of medications not just for glucose-lowering but also for weight reduction and for their evidence base in cardiovascular and renal risk reduction [3]. In contrast, there remains both ambiguity and inertia in recognizing and addressing metabolic dysfunction in type 1 diabetes. In this narrative review, we set out the case for a shift in our thinking about type 1 diabetes management and risk mitigation of long-term cardiorenal disease and multimorbidity. We discuss current shortfalls in risk stratification in type 1 diabetes, the evidence base for the use of the major classes of adjunct therapies (originally developed and trialled for type 2 diabetes), and suggest future research priorities. 

**Box 1:**
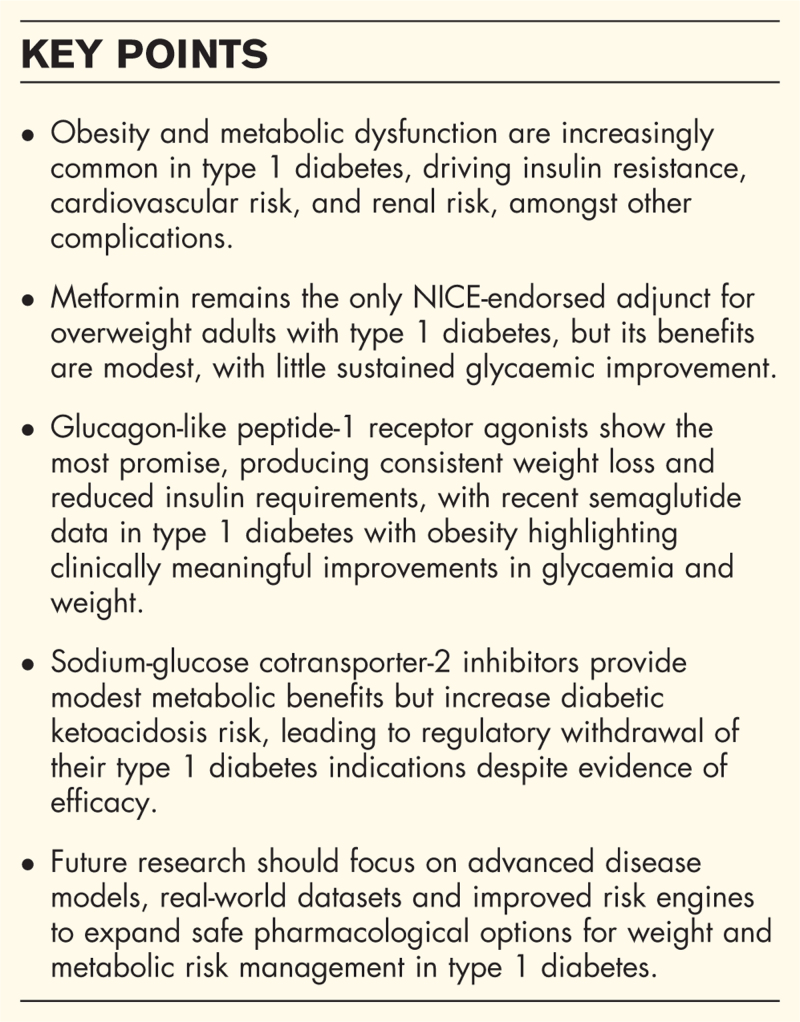
no caption available

## THE RISING INCIDENCE OF OBESITY-ASSOCIATED METABOLIC DYSFUNCTION IN PEOPLE LIVING WITH TYPE 1 DIABETES

In the past, type 1 diabetes has traditionally been considered a disease of lean people [4]. However, in recent years, the reported occurrence of overweight and obesity in the type 1 diabetes population has prompted a re-evaluation [5]. Temporal patterns have suggested that increases in the prevalence of overweight and obesity in the type 1 diabetes population have far outpaced that in the general population [6]. In the United Kingdom, it is estimated that ~60% of people with type 1 diabetes also live with overweight or obesity, and similarly, in the United States, a study analysing adult type 1 diabetes data from the National Health Interview Survey between 2016 and 2021 found the prevalence of obesity and overweight to be 28% and 34%, respectively [7,8].

It is often noted that a potential driver for weight gain in type 1 diabetes is insulin therapy itself. In the Diabetes Control and Complications Trial, significant weight gain was found in both the intensive and standard (1–2 insulin injections per day) insulin treatment groups [9]. Comparatively, the intensive treatment group experienced higher weight gain, with ~25% becoming obese [10]. Herein, a vicious cycle is created, as people with type 1 diabetes and overweight/obesity need larger doses of insulin to achieve the same level of glycaemic control as those with a BMI in a healthy range [11]. Insulin-associated weight gain, particularly in those with suboptimal glycaemic control, is probably largely due to improved fuel utilization/reduced renal glucose losses without a concomitant reduction in calorie intake. It may also be caused by the “unphysiological” pharmacokinetic profile of subcutaneously delivered insulin – for example, an unconscious increase in calorie intake caused by the fear or experience of hypoglycaemia [4]. However, recent developments in modern, ultra-rapid analogue insulins have shown no evidence of less associated weight gain, and although insulin pump therapy may result in lower total daily doses (TDD) of insulin, there is no emerging evidence to suggest that this results in less weight gain. In essence, the prevalence of overweight and obesity has now become similar in people with type 1 diabetes and the general population, and more research is needed to understand how best to mitigate weight gain and advise on weight loss interventions for people living with type 1 diabetes.

As the prevalence of obesity is rising in type 1 diabetes, so is the association with broader metabolic morbidity. A real-world study of 27 000 adults with type 1 diabetes demonstrated that although glycaemic and lipid control improved between 2001 and 2022, the prevalence of combined metabolic dysfunctions, known as the metabolic syndrome, sharply increased from 12.1% to 21.7% [12]. There have been attempts to define the metabolic dysfunction and its related co-morbidities in type 1 diabetes from organizations such as the International Diabetes Federation and the American Heart Association [13]. Since dysglycaemia is preexisting, most definitions revolve around the additional presence of central adiposity and dyslipidaemia. Insulin resistance is acknowledged as an important pathogenic driver of the increased incidence of cardiovascular complications that occurs in people living with both type 1 diabetes and metabolic dysfunction [14] and, whilst some clinicians may turn to units/kg of insulin dosed as a guide, there is no agreed way of measuring it. In summary, the lack of consensus on a standardized criteria makes it difficult for clinicians to actively diagnose insulin resistance and metabolic dysfunction in people living with type 1 diabetes [12].

The presence of obesity and the metabolic syndrome is associated with an accelerated risk of diabetes-related complications, such as cardiovascular and chronic kidney disease, or peripheral neuropathy over and above that caused by dysglycaemia alone in people with type 1 diabetes [12,14]. Risk-stratification for these complications is essential. A rule-based approach is often used based on assessing inflection points for increased age-related risk – for example, the UK National Institute for Health and Care Excellence (NICE) recommends statins for primary prevention in type 1 diabetes patients who are ≥40 years old, have had diabetes ≥10 years, have nephropathy, or have additional cardiovascular risk factors. The QRISK3 engine, although not developed for people living with type 1 diabetes, has also been assessed to be useful in this patient group [15]. In a consensus report by the American Diabetes Association and the European Association for the Study of Diabetes, it is similarly recommended that statins should be considered in those over 40 years old, those with increased atherosclerotic cardiovascular disease risk factors aged 20–39 years old, or when the 10-year cardiovascular risk (as calculated by a suitable risk engine) exceeds 10% [16]. As noted in the report, the recommendations rely on guidelines for type 2 diabetes given the lack of relevant trials in people with type 1 diabetes.

## INSULIN ADJUNCTS

Recently, medications traditionally used in type 2 diabetes have been prescribed as adjuncts to insulin in type 1 diabetes to address glycaemic control, obesity, and metabolic dysfunction [17]. It is highly likely that adjunct medications will be beneficial to subset populations of those living with type 1 diabetes, but who will benefit the most, at what stage and whether they can be treated safely, is poorly understood. Studies show that those who have been prescribed adjuncts off-licence typically have elevated cardiorenal risk profiles compared to the general type 1 diabetes population – other common factors determining use include weight, and high insulin dose [17,18]. Clinical decisions to use these medications “off-licence” are usually informed by extrapolated evidence from studies in type 2 diabetes, but with limited evidence base and safety data in the type 1 diabetes population itself. Therefore, these treatments are not widely endorsed in guidelines for type 1 diabetes management. This omission is partly due to concerns that some drugs designed for type 2 diabetes (e.g. SGLT-2 inhibitors) may pose risks, such as diabetic ketoacidosis (DKA), in the context of the absolute insulin deficiency of type 1 diabetes [19]. Additionally, conducting adequately powered cardiovascular outcomes trials in younger type 1 diabetes populations poses significant challenges as event rates are lower, and therefore high-quality evidence to inform guidelines is unlikely to be forthcoming. In the next sections we explore new concepts in the definition of metabolic dysfunction, and review the current data for the use of the three major classes of adjuncts in type 1 diabetes that have the best evidence base for cardiovascular benefits.

## BREAKTHROUGH CONCEPTS IN METABOLIC MULTIMORBIDITY

In recency, two important consensus papers have been published, expounding the development of multiorgan disease in the setting of metabolic dysfunction. The first, a scientific statement from the American Heart Association, describes the cardiovascular-kidney-metabolic (CKM) syndrome [20^▪^]. This concept has developed from an improved understanding of the complex, multidirectional interplay between metabolic risk factors, chronic kidney disease (CKD) and cardiovascular disease (CVD), noting that many new medications are evidenced to impact on all three. The authors begin with the driving features of dysfunctional, excess adipose tissue. However, compared with previous descriptions of the metabolic syndrome, there is much more focus on reciprocal risk amplification and the centrality of renal dysfunction. For example, there is a well known increased risk of CVD from the very earliest stages of renal endothelial dysfunction (microalbuminuria) whilst frank CKD, especially when present with diabetes, is a proinflammatory state that accelerates atherosclerosis. When discussing the major classes of drugs trialled in this setting, we find a striking difference in the way in which ACE inhibitors are widely adopted in type 1 diabetic kidney disease whilst agents like SGLT-2 inhibitors and fineronone remain under-utilized. The CKM authors note the independent effects of hyperglycaemia per se on endothelial dysfunction and the acceleration of both CKD and CVD risk, yet stop short of incorporating T1D into their discussion. Moves towards lifelong, longitudinal screening and restaging for CKM, with data-driven risk stratification particularly serving populations typically underrepresented in clinical trials are advocated. We would encourage populations with T1D to be included in that.

In the second paper, Godoy-Matos *et al.* emphasize that recent frameworks do not focus enough on the role of metabolic dysfunction-associated steatoic fatty liver disease (MASLD) in multisystem morbidity [21^▪^]. The proposed CARDIAL-MS (Cardio-Renal-DIAbetes-Liver-Metabolic Syndrome) model extends and expands the cardio-kidney-metabolic syndrome framework, integrating MASLD as an additional core component. Current evidence shows that MASLD and diabetes are linked to both cardiovascular and renal diseases, and the liver crucially contributes to key processes that define metabolic syndrome such as excessive fasting hepatic glucose output, abnormal insulin clearance, and inflammation. There is limited data describing the issue of liver-related metabolic dysfunction in the context of type 1 diabetes. A systematic review and meta-analysis from 2020 (*n* = 3901) found the pooled MASLD (formerly known as nonalcoholic fatty liver disease) prevalence to be 22.0% amongst adults living with type 1 diabetes, though this estimate is highly contingent on the mode of imaging and diagnostic criteria employed [22]. There is large heterogeneity in prevalence between recent studies – using ultrasound, albeit with differing diagnostic criteria, Mertens *et al.* (2023, *n* = 530) reported a MASLD prevalence of 16.2%, whereas Vergani *et al.* (2025, *n* = 198) found a prevalence of 37.0% [23,24]. Mertens *et al.* found MASLD was associated with both metabolic syndrome and obesity, and Vergani *et al.* identified an inverse association between MASLD and estimated glucose disposal rate, a reliable, noninvasive surrogate of insulin sensitivity. Despite an uncertainty over the true picture of the problem, the association between liver dysfunction, the metabolic syndrome, and type 1 diabetes is clear, and the implementation of screening is necessary to treat this largely forgotten and underserved population.

## METFORMIN

The only adjunct to insulin recommended by NICE in type 1 diabetes is metformin, a biguanide commonly used in treating type 2 diabetes, though its use in the former remains unlicensed [25]. It is recommended for adults with type 1 diabetes and a BMI greater than 25 kg/m^2^ (23 kg/m^2^ for people from South Asian and related minority ethnic groups) to improve their blood glucose control while minimizing insulin doses. Metformin is thought to work by decreasing hepatic glucose production and increasing insulin sensitivity. Meta-analyses of its use in type 2 diabetes reveal 1–2% reduction in HbA1C and significant reductions in cardiovascular events and mortality [26].

There are far fewer trials of metformin in type 1 diabetes. Over three years, the REMOVAL trial found metformin to have no sustained improvement in glycaemic control, and only a modest effect in weight reduction [−1.17 kg, 95% confidence interval (CI) −1.66 to −0.69] in people with type 1 diabetes [27]. In other secondary outcomes, reductions were observed in measures such as insulin dose requirement per unit of bodyweight (visit-by-treatment interaction *P* = 0.0018), and low-density lipoprotein cholesterol (−0.13 mmol/mol, 95% CI −0.24 to −0.03). The primary outcome, which was atherosclerosis progression as measured by the progression of mean common carotid artery intima media thickness, was also not significantly reduced, though there was a reduction in the prespecified tertiary outcome of averaged maximal common carotid artery intima media thickness.

New research also calls into question the effect of metformin on insulin resistance. Amongst adults with type 1 diabetes, a 26-week trial of metformin against placebo found no difference in the primary outcome of change in endogenous glucose production during the low-dose phase of a two-step hyperinsulinaemic–euglycaemic clamp – the gold-standard technique to measure insulin resistance [28]. Compared to placebo, metformin significantly reduced total daily insulin dose by 0.10 units/kg/day, a commonly used surrogate for insulin resistance, but there were no differences found in other secondary outcomes such as HbA1c or glycaemic variability. The reduction in insulin dose without a change in endogenous glucose production suggests that metformin may have metabolic effects which spare insulin requirements independent of its effects on suppressing gluconeogenesis.

Currently, metformin is commonly prescribed as an adjunct in type 1 diabetes – 94% of respondents to an online survey of Australian endocrinologists stated they had prescribed the drug previously, and as of 2016, 15% of adults in Scotland living with type 1 diabetes have received metformin [18,29]. It is generally regarded as a safe drug, with a small caveat in that it may increase the incidence of gastrointestinal side effects and B12 deficiency, though neither of which obviate the clear cardioprotective and weight benefits [29,30].

## GLUCAGON-LIKE PEPTIDE-1 RECEPTOR AGONISTS

Glucagon-like peptide-1 receptor agonists (GLP-1RAs) have direct anorectic effects, achieved by engaging the glucagon-like peptide-1 receptors in the appetite centres of the brain, and by slowing gastric emptying postprandially [31]. For those with residual β-cell function, they also increase insulin secretion in a glucose-dependent manner [32,33^▪^,^34^]. Additionally, GLP-1RAs suppress α-cell secretion of glucagon, which in turn decreases inappropriate postprandial hyperglycaemia and glycaemic variability [32]. However, as the same mechanism can also increase the risk of hypoglycaemia, concerns over safety have been posed.

Given their nonglycaemic benefits, GLP-1RAs have become increasingly commonplace, with drugs like semaglutide being licensed for use in obesity without diabetes [35–37]. In SUSTAIN-6, semaglutide reduced major adverse cardiovascular events (MACE) in patients with type 2 diabetes by 26% compared to placebo, and in FLOW, semaglutide significantly reduced eGFR decline from baseline in type 2 diabetes patients with chronic kidney disease (CKD) [38]. Given the very strong evidence base for their benefits in terms of cardiorenal risk reduction, GLP-1RAs are now advocated as first-line treatment in patients with type 2 diabetes and atherosclerotic cardiovascular disease, and are generally approved for type 2 diabetes in countries such as the United States and Japan [3,39,40]. Despite showing clear benefits in people with type 1 diabetes, worries persist over side effects such as DKA and hypoglycaemia and very low representation of patients with type 1 diabetes in GLP-1RA trials have prevented these drugs from being licensed for type 1 diabetes [19,29,41].

Early trials of GLP-1RA in type 1 diabetes were not resoundingly positive. The ADJUNCT ONE and ADJUNCT TWO trials were published in 2016, testing the much earlier GLP-1RA liraglutide [42,43]. In the ADJUNCT trials, liraglutide was reported to cause a higher number of hypoglycaemia, hyperglycaemia and ketoacidosis events and, as such, the FDA considered it to be insufficiently safe for patients [44]. However, glycaemic recovery, counterregulatory responses, and gastric emptying during hypoglycaemia in type 1 diabetes have shown to be unaffected by liraglutide, providing reassurance that shorter-acting GLP-1RAs do not impair physiological responses to hypoglycaemia [45]. Additionally, since the ADJUNCT trials, the use of diabetes technologies, such as continuous glucose monitoring (CGM) and AID, have become far more commonplace. These technologies have the capability of reliably identifying hypoglycaemia or hyperglycaemia, and, in the case of hybrid closed loop systems, correcting such glycaemic variability. Furthermore, newer GLP-1RAs, such as semaglutide, have increased weight loss potency and an ever-increasing evidence base for their cardiovascular benefits. Larger clinical trials of modern GLP-1RAs in type 1 diabetes, specifically in populations using CGM or AID, are needed to demonstrate that GLP1-RAs can be safe for patients to use, and that the potential benefits in weight, insulin dose, and glycaemic control can outweigh the potential safety concerns when sufficient precautions are taken.

In this light, more recent, small randomized controlled trials present strong evidence supporting the use of GLP-1RAs in tackling obesity and glycaemic control in type 1 diabetes. Shah *et al.* conducted a 26-week multicentre, double-blind, placebo-controlled trial of semaglutide in 72 patients with obesity and type 1 diabetes using an automated insulin delivery system [46^▪^]. At 26 weeks, a significantly greater percentage of the semaglutide group achieved the primary composite outcome (36% vs. 0%) of ≥70% time in glucose sensor range of 70–180 ml/dl, ≤4% time below glucose sensor range of 70 mg/dl, and 5% reduction in body weight. The semaglutide group experienced 8.8 kg weight reduction compared to the placebo group, and saw a clinically significant reduction of 22.3 units/day in total daily insulin use. Severe hypoglycaemia was reported in two patients in both groups, and there were no reports of DKA. Only two patients in the semaglutide group discontinued due to persistent gastrointestinal adverse events. Another double-blind, crossover trial testing weekly subcutaneous semaglutide in a patient population using AID found that compared to placebo, semaglutide significantly increased time in target glucose range (3.9–10.0 mmol/l), without increasing the time below 3.9 mmol/l or 3.0 mmol/l [47^▪^]. Similarly, no cases of DKA or severe hypoglycaemia were observed. These results support the use of semaglutide as a safe and tolerable adjunctive therapy. The reduced TDD is a worthy take home message since this may help patients become eligible for pumps with lower insulin cartridge capacity.

The results and conclusions of these developments are reflected in a recent consensus report on the use of GLP-1RAs as adjunctive treatment for individuals using AID. The expert opinion, agreed upon by a panel comprised of endocrinologists and engineers mainly from the United States, but also Europe, is that GLP-1RAs could provide an effective means of improving both metabolic and glycaemic outcomes, without increasing the risk of severe hypoglycaemia and DKA [44]. The report strongly recommends GLP-1RA-based therapies for specific populations, such as adults with insulin resistance, high insulin requirement, or overweight or obesity, who cannot achieve optimal glycaemic control despite intensive therapy, or those who experience postprandial hyperglycaemia despite meal plan optimisation. A recent real-world study of 125 adult type 1 diabetes patients from the Diabetes Prospective Follow-up Registry found that GLP-1RAs are safe and largely tolerable, with no significant increase in DKA or severe hypoglycaemic events. However, it also noted that over an average treatment duration of 1.9 years, there were no significant effects on HbA1c weight, blood pressure, cholesterol, or MACE [48]. It is therefore clear that patient selection is an important consideration as we move towards a consensus on the use of GLP-1RAs in type 1 diabetes. There is limited evidence to date about which baseline characteristics can help optimize patient selection for GLP-1RA use, in terms of predictors of drug efficacy (for glycaemia as well as cardiovascular endpoints) and tolerability. It has been suggested that patients with remaining C-peptide tend to have the largest glycaemic improvement from GLP-1RA use, aligning with other studies [44,47^▪^,^49^]. Posthoc analysis of the ADJUNCT ONE and ADJUNCT TWO studies found that patients with lower levels of C-peptide at baseline and longer diabetes disease duration were more likely to discontinue liraglutide treatment due to drug intolerance or adverse events than patients with residual β-cell function [50]. Taken together, there is increasing evidence for the safe and effective use of modern GLP-1RAs in type 1 diabetes, coupled with increasing patient demand, and an urgent need to identify which subpopulations have most to gain. This includes patients with diabetic foot disease and neuropathy as well as those with elevated cardiovascular risk due to obesity or the metabolic syndrome.

## DUAL GUT HORMONE RECEPTOR AGONISTS

Tirzepatide works as a dual GLP-1/glucose-dependent insulinotropic polypeptide (GIP) receptor agonist, developed to treat type 2 diabetes and obesity. In patients with obesity without diabetes, tirzepatide was superior to semaglutide in reducing total body weight after 72 weeks (20.2% vs. 13.7%) [51]. In type 2 diabetes, a meta-analysis of 15 GLP-1RAs found tirzepatide to be the most effective drug for glycaemic control, and second only to CagriSema (semaglutide with cagrilintide) in inducing weight loss [52]. However, compared to GLP-1RAs, there are currently fewer studies examining tirzepatide's effects on long-term cardiovascular and renal outcomes, though limited trial data suggests it will at least match the benefits of GLP-1RAs alone, if not surpassing them [53].

TIRTLE1, the first phase 2 randomized placebo-controlled trial of tirzepatide in adults with type 1 diabetes, found that after 12 weeks, tirzepatide significantly reduced weight (estimated treatment difference −8.7 kg [95% CI −12.0 to −5.5 kg]), HbA1c (mean difference −0.4% [95% CI −0.7 to 0.0%]), and insulin use (mean difference −35.1%, 95% CI -46.5 to −21.3%) compared to placebo [54^▪^]. All participants in the tirzepatide group experienced clinically significant weight loss >5%, with 45% of the participants experiencing >10% weight loss. In the placebo group, the single participant (9%) that experienced >5% weight loss restricted calorie intake and increased physical activity. The drug was also well tolerated – there were no serious adverse events, episodes of DKA, or severe hypoglycaemia. According to a self-reported questionnaire, the only between-group difference was a significant increase in fullness and early satiety vs. baseline in the tirzepatide group. Several more phase 2 and 3 trials, such as TZP-T1D and the SURPASS-T1D-1 and -2 trials, are also currently underway [55–57].

Retrospective cohort studies note significant weight loss and improvement in glycaemic control, though small sample sizes and confounding factors limit any conclusions made [58,59^▪^]. Randomized controlled trials or large-scale real-world analyses are needed to further evaluate its safety in type 1 diabetes and efficacy in terms of glycaemia benefits but also longer term cardio-renal risk reduction.

## SODIUM-GLUCOSE COTRANSPORTER-2 INHIBITORS

Sodium-glucose cotransporter-2 inhibitors (SGLT2i) originally ascended in the type 2 diabetes treatment algorithm due to their independent nonglycaemic benefits, namely improvements in cardiovascular outcomes and renal protection (see Table 1). In EMPA-REG OUTCOME, empagliflozin reduced the risk of cardiovascular death by 38% relative to placebo in patients with type 2 diabetes at high cardiovascular risk [60]. In the CREDENCE study, canagliflozin also reduced the relative risk of the primary outcome, a composite of end-stage kidney disease, doubling of creatinine, or death from renal/cardiovascular causes, by 30% compared to placebo in patients with type 2 diabetes and chronic kidney disease [61]. Like empagliflozin, canagliflozin was also associated with a lower risk of myocardial infarction, stroke, and cardiovascular death. In the DECLARE trial, dapagliflozin, which is commonly used in type 2 diabetes, similarly lowered the rate of cardiovascular death or hospitalization for heart failure compared to placebo in type 2 diabetes patients who previously had, or were at risk of, atherosclerotic cardiovascular disease [62]. However, it had no significant effect on the rate of major adverse cardiovascular events. Whilst there is no compelling evidence of the superiority of one SGLT2i over another, only dapagliflozin and empagliflozin are licensed for patients with eGFR <30 in the UK [63].

**Table 1:** Summary of clinical trials establishing the cardiovascular and renal benefits of SGLT2 inhibitors

Trial	Drug (dose)/Comparator	Population (n)	Median Follow-Up Period	Key Cardiovascular/Renal Findings	Relative Risk Reduction	Notes
EMPA-REG OUTCOME [60]	Empagliflozin (10mg or 25mg OD)/placebo	T2D with established CVD (7,020)	3.1 years	↓risk of CV death ↓hospitalisation for HF ↓all-cause mortality	38% 35% 32%	Proportion of patients with confirmed DKA similar across groups
CREDENCE [61]	Canagliflozin (100mg OD)/placebo	T2D with CKD (4,401)	2.62 years	↓composite primary outcome (ESKD, doubling of creatinine, renal/CV death) ↓risk of MI, stroke, or CV death	30% 20%	Stopped early after interim analysis
DECLARE [62]	Dapagliflozin (100mg OD)/placebo	T2D with/at risk of atherosclerotic CVD (17,160)	4.2 years	↓CV death, hospitalisation for HF	17%	No significant effect on rate of MACE Only 40% of participants had established CVD – 60% primary prevention No between-group difference in CV death

CKD, chronic kidney disease; CV, cardiovascular; CVD, cardiovascular disease; DKA, diabetic ketoacidosis; ESKD, end-stage kidney disease; HF, heart failure; MACE, major adverse cardiovascular event; MI, myocardial infarction; OD, once daily; T2D, type 2 diabetes.

There are several mechanisms involved in these effects. By blocking the SGLT2 co-transporter in the proximal convoluted tubule, glucosuria and mild osmotic diuresis are caused [64]. Through this, tubuloglomerular feedback is activated, and intraglomerular pressure is reduced – a central role in renal protection. Additional mechanisms such as natriuresis, leading to reduced intravascular volume, preload, and blood pressure, as well as reduced low-grade inflammation and oxidative stress, are all thought to play a role in cardiovascular benefits beyond improved glycaemic control [64,65]. Moreover, SGLT2i can induce weight loss through numerous mechanisms centred around lipolysis and the loss of excess glucose in the urine, which also helps to improve glycaemic control [66].

Given their success in type 2 diabetes, trialling SGLT2i in type 1 diabetes was a natural progression. Dapagliflozin was authorized in 2019 in the UK as an insulin adjunct for type 1 diabetes patients with a body-mass index of 27 kg/m^2^ or above, for whom, despite optimization, insulin did not provide adequate glycaemic control [67]. Though it was eventually withdrawn in 2021 from both the UK and across Europe, the drug is still licensed for type 1 diabetes in countries such as Japan [68]. The decision was made voluntarily by the manufacturer, and was not due to new safety concerns – the increased risk of DKA was already known prior to the drug's authorization. However, the relatively high frequency of DKA was still noted, with some studies in type 1 diabetes reporting up to 1 in 10 patients to be affected, and currently no SGLT2i are licensed for use in type 1 diabetes in the UK or the United States. The exact pathogenesis behind (often euglycaemic) DKA in SGLT2i is not well understood, though several mechanisms have been proposed [69]. For example, SGLT2i increase α-cell secretion of glucagon, which can inhibit acetyl-CoA carboxylase and promote carnitine palmitoyltransferase-I activity, leading to subsequent ketogenesis. In the kidneys, a reduction in renal clearance of ketones/ketonuria may also contribute to DKA.

With a caution about DKA in mind, there is a renewed interest in the use of SGLT2i in type 1 diabetes because of the growing evidence base of their independent cardiorenal benefits. A 2024 meta-analysis of 16 randomized controlled trials with 7192 patients with type 1 diabetes found mean HbA1c reductions of 0.29%, with decreases improving gradually over time [70]. Weight loss was also noted, most evident after 2–6 months of treatment (mean difference: −4.73 kg). Though weight loss plateaued in subsequent periods, these effects remained stable for up to 12 months of treatment. Significant reductions in total daily dose of insulin were also shown, with a mean difference of −7.14 units/day between groups – a key benefit given the association of insulin use with weight gain. Of the 7 studies included in the DKA analysis, it was shown that SGLT2i were overall related to a higher risk compared to placebo (risk ratio = 1.44), but there was no increased risk observed within the first month of treatment.

To address the problem of DKA, dapagliflozin and glucagon receptor antagonist combination therapy was tested in a recent randomized, placebo-controlled crossover trial consisting of 12 patients using AID [71^▪^]. In type 1 diabetes, SGLT2i therapy has been associated with a 37% increase in fasting glucagon levels, driving excess endogenous glucose production and, especially in insulin-deficient states, increased ketogenesis. During insulin withdrawal tests, peak β-hydroxybutyrate (a key ketone body) was 17% lower (*P* = 0.048) for the combination therapy group than the SGLT2i-only group, demonstrating a potential solution to DKA – one of the largest obstacles to SGLT2i therapy in type 1 diabetes. Additionally, over 4 weeks, combination therapy significantly improved glycaemic control, increasing time in range by 8% compared to SGLT2i-only therapy, and by 16% compared to baseline insulin-only measurements. Total daily insulin was also the lowest in combination therapy at 0.41 units/kg/day, compared to 0.52 and 0.56 units/kg/day for SGLT2i-only and baseline. Despite its modest sample size and treatment duration, the study importantly shows the potential of SGLT2i and glucagon receptor antagonist combination therapy in type 1 diabetes, and provides a direction for larger-scale trials in the future.

The same real-world GLP-1 study of the Diabetes Prospective Follow-up Registry also examined the effectiveness of SGLT2i [48]. After an average treatment duration of 2.5 years, there was no significant difference in weight or body mass index before or after initiation. However, HbA1c showed a significant decrease of 0.3%, alongside significant reductions in systolic blood pressure, total cholesterol, and low-density lipoprotein cholesterol, all without an increased risk of severe hypoglycaemic or DKA. The authors noted that compared with an earlier timepoint analysis of the cohort (at 12 months postinitiation), the mean reductions in HbA1c, blood pressure, and total cholesterol were abrogated, suggesting benefits may diminish over time. There remains insufficient trial evidence to be sure that the same protective cardiorenal effects of SGLT2i exist in type 1 diabetes as they do in type 2 diabetes.

Finally, it is worth noting that SGLT2i have a clear evidence base in the setting of both ejection fraction reduced and ejection fraction preserved heart failure [72–74]. Therefore, this class of drug is being increasingly used for that indication in patients with co-existing type 1 diabetes. Whilst it seems sensible to assume that the independent cardioprotective benefits should apply to patients with type 1 diabetes, it is incumbent to ensure that patients are supported with insulin doses titration around initiation, and are very well educated around ketone monitoring, sick day rules, and when to discontinue the SGLT2i [75,76].

## CLOSING REMARKS

There is growing recognition of overlapping phenotypes between type 1 and type 2 diabetes with an acceptance for the need to recognize dysmetabolic parameters and reduce the risk of longer term cardiovascular multimorbidity in people living with type 1 diabetes. There has been a rapid expansion in this century in classes of drugs available to treat type 2 diabetes, with increasing evidence of their pleotropic cardiorenal benefits in addition to glucose lowering. However, cardiovascular disease is also the leading cause of death in people living with type 1 diabetes [77]. Age and duration of type 1 diabetes are obviously important, but with a paucity of research defining metabolic dysfunction in type 1 diabetes for detailed risk stratification in this group, there is less certainty about which patients with type 1 diabetes will benefit most from which adjunct and when [78].

The off-licence use of adjuncts for people living with type 1 diabetes is already increasing rapidly, particularly amongst specialist prescribers with expertise in diabetes and diabetic renal disease [17]. For this trend to become established equitably, and to target those with most to benefit, whilst minimizing risks, we propose the following strategies to embed an evidence base for the use of adjuncts in type 1 diabetes. Randomized controlled trials of course must play a role, with a push to include people living with type 1 diabetes in future ones. Historically, trials of adjuncts in type 1 diabetes have focused on glycaemic outcomes, though emerging evidence suggests that the metabolic burden extends beyond that, encompassing weight, total daily insulin dose, and long-term cardiorenal risks. Adopting broader outcomes that focus on these factors, alongside patient-reported outcomes such as quality of life and treatment satisfaction, would help more fully determine the value of adjunctive therapy. Given the limitations of type 1 diabetes representing only 10% of diabetes, and the requirement for longer follow up times, other approaches must also be adopted. Real-world data sets offer the opportunity to mine national clinical registries to begin to define cardiometabolic features in people living with type 1 diabetes and stratify their onset with future risk. Patient derived cell lines and advanced organ on chip systems to interrogate endothelial dysfunction offer the opportunity to model disease and drug responses for vascular complications in T1D directly [79]. These novel research methods lay the foundation for improved disease modelling, biomarker identification, risk engines and patient selection for targeted trials.

## ACKNOWLEDGEMENTS

*None.*

### Financial support and sponsorship

*S.M. is funded by a Wellcome Trust Career Development Award [223024/Z/21/Z], Breakthrough Type 1 Diabetes, and is supported by the NIHR Imperial Biomedical Research Centre. V.S. is funded by Diabetes UK.*

### Conflicts of interest

*S.M. has received speaker honoraria from Lilly, Sanofi and Menarini.*

## REFERENCES AND RECOMMENDED READING

Papers of particular interest, published within the annual period of review, have been highlighted as:

▪ of special interest
▪▪ of outstanding interest
